# Prevalence of Lynch Syndrome among Patients with Newly Diagnosed Endometrial Cancers

**DOI:** 10.1371/journal.pone.0079737

**Published:** 2013-11-07

**Authors:** Cecilia Egoavil, Cristina Alenda, Adela Castillejo, Artemio Paya, Gloria Peiro, Ana-Beatriz Sánchez-Heras, Maria-Isabel Castillejo, Estefanía Rojas, Víctor-Manuel Barberá, Sonia Cigüenza, Jose-Antonio Lopez, Oscar Piñero, Maria-Jose Román, Juan-Carlos Martínez-Escoriza, Carla Guarinos, Lucia Perez-Carbonell, Francisco-Ignacio Aranda, Jose-Luis Soto

**Affiliations:** 1 Pathology, Alicante University Hospital, Alicante, Spain; 2 Research Laboratory, Alicante University Hospital, Alicante, Spain; 3 Molecular Genetics Laboratory, Elche University Hospital, Elche, Spain; 4 Genetic Counselling in Cancer Unit, Elche University Hospital, Elche, Spain; 5 Gynecology, Alicante University Hospital, Alicante, Spain; Sapporo Medical University, Japan

## Abstract

**Background:**

Lynch syndrome (LS) is a hereditary condition that increases the risk for endometrial and other cancers. The identification of endometrial cancer (EC) patients with LS has the potential to influence life-saving interventions. We aimed to study the prevalence of LS among EC patients in our population.

**Methods:**

Universal screening for LS was applied for a consecutive series EC. Tumor testing using microsatellite instability (MSI), immunohistochemistry (IHC) for mismatch-repair (MMR) protein expression and *MLH1*-methylation analysis, when required, was used to select LS-suspicious cases. Sequencing of corresponding MMR genes was performed.

**Results:**

One hundred and seventy-three EC (average age, 63 years) were screened. Sixty-one patients (35%) had abnormal IHC or MSI results. After *MLH1* methylation analysis, 27 cases were considered suspicious of LS. From these, 22 were contacted and referred for genetic counseling. Nineteen pursued genetic testing and eight were diagnosed of LS. Mutations were more frequent in younger patients (<50 yrs). Three cases had either intact IHC or MSS and reinforce the need of implement the EC screening with both techniques.

**Conclusion:**

The prevalence of LS among EC patients was 4.6% (8/173); with a predictive frequency of 6.6% in the Spanish population. Universal screening of EC for LS is recommended.

## Introduction

Identification of hereditary forms of neoplasias among cancer patients is crucial for better management and prevention of other syndrome-associated malignancies for the patients and their families [1]. The estimated incidence and mortality count of endometrial cancer (EC) in Europe in 2012 were 58,300 and 24,400, respectively. EC accounts for approximately 4% of all cancers in women [2]. The incidence is increasing and approximately 5% of cases are thought to result from a genetic predisposition [3].

Lynch syndrome (LS) is an autosomal dominant condition caused by a mutation in the mismatch repair (MMR) genes, *MLH1*, *MSH2*, *MSH6* and *PMS2* [4]. Mutation carriers are at risk of early onset colorectal cancer (CRC), EC and a spectrum of other tumors such as ovarian, gastric, small bowel, pancreatic, hepatobiliary, brain and urothelial neoplasms [5]. The cumulative lifetime risk of EC for female carriers of an MMR mutation is 50–60% and exceeds the risk of a CRC [6].

The identification of patients with EC and LS has the potential to influence life-saving interventions through personalized counseling and intensive cancer surveillance with early detection, screening and prevention of other LS-associated cancers. Genetic testing is now an accepted part of the management of patients with such cancers. The Mallorca group [7] recommended testing all cases of CRC (or individuals with a CRC aged <70 years) and all cases of EC (or individuals with an EC aged <70 years) by immunohistochemistry (IHC) for MMR genes or chromosomal microsatellite instability (MSI).

The prevalence of LS among unselected cases of CRC has been studied well. The results indicate that 0.7% to 3.6% of all such cases might be caused by germline mutations in MMR genes [8–12]. On the contrary, research on LS-related ECs is still evolving and little is known about the genetic components among patients with EC. Current data on the prevalence of LS among unselected cases of EC in North America range between 1.8% and 4.5% [13–15]. Significant differences in the prevalence of hereditary syndromes are frequently observed among different populations [12]. Here we report on the prevalence of LS in a consecutive series of patients with EC from the Spanish population.

## Materials and Methods

### Ethical issues

Tumor tissue and blood samples from patients with EC were obtained from the Biobank of the Alicante University Hospital (HGUA) in Spain. Written consent to be included in the Biobank was obtained from each patient. The Ethics Committee of HGUA approved the study.

### Subjects

One hundred seventy-three consecutive patients with newly diagnosed ECs were included in this study. They were diagnosed and treated at the HGUA from 2004–2009. For each case, all available hematoxylin–eosin slides were reviewed and classified using the 2003 World Health Organization criteria [16]. Additional histopathology features recorded were the presence of lymphovascular invasion (LVI), tumor-infiltrating lymphocytes (TILs), myometrial invasion, and grade and stage according to the International Federation of Obstetrics and Gynaecology (FIGO). The adenocarcinomas were classified into endometrioid (type I) and special (type II) according to Peiró et al 2013 [17]. A familial history of cancer was evaluated according to the revised Bethesda guidelines (rBG) and Amsterdam II criteria (AmII) [18].

### Immunohistochemistry of MMR proteins

IHC analysis of the expression of the MLH1, MSH2, MSH6 and PMS2 proteins was performed using a tissue microarray (TMA). Tissue cylinders of 1 mm diameter were punched out from selected areas and incorporated into a recipient paraffin block using a TMA instrument (MTA-1,Beecher Instrument, Wisconsin USA). Four-millimeter-thick sections were prepared from the TMA samples. The slides were placed on a Autostainer Link48 (Dako, Denmark) and incubated for 30 min at room temperature with primary antibodies to MLH1 (clone G168–15; 1:30; BD Biosciences Pharmingen, Franklin Lakes, NJ, USA), MSH2 (clone 44; dilution 1:100; BD Transduction Laboratories, San Diego, CA, USA), MSH6 (clone FE11; dilution 1:30; Calbiochem, Merck Millipore, Billerica, MA, USA) and PMS2 (clone A16–4; dilution 1:100; BD Biosciences Pharmingen). Antibodies were detected using the EnVision technique (Dako-Biotech). Tumor cells were judged as negative for protein expression only if they lacked IHC staining in a sample in which normal endometrial cells and stromal cells were stained concurrently. Results were considered unreliable in those cases where no immunostaining of normal tissue could be demonstrated. The processed IHC slides were blinded evaluated by two pathologists (CE and CA) [19].

### DNA extraction

Genomic DNA was isolated from paraffin wax-embedded tumor tissues. Two 1 mm tissue cylinders were punched out from the tumor areas selected previously. DNA from peripheral blood leukocytes or from paraffin wax-embedded nontumorous endometrium tissue was also extracted from those cases where LS was suspected. A DNeasy Blood & Tissue kit and QiaCube (Qiagen, Valencia, CA, USA) automatic system was used to isolate DNA, according to the manufacturer’s protocol.

### Microsatellite analysis and MMR status

MSI status was analyzed using multiplexed polymerase chain reaction (PCR) patterns at the monomorphic repetitive markers: BAT26, BAT25, NR21, NR24 and NR27 [20]. Amplicon detection and analysis were performed using an ABI Prism 3130 Genetic Analyzer, and Genotyper software (Life Technologies, Carlsbad, CA, USA), respectively. A diagnosis of MSI was considered positive when two or more markers showed an altered pattern. Tumors with MSI and/or loss of expression of any of the MMR proteins were considered as MMR-deficient. Tumors with absence of MSI and preserved MMR protein expression were considered as MMR-positive tumors.

### *MLH1* promoter hypermethylation analysis

**Figure 1:**
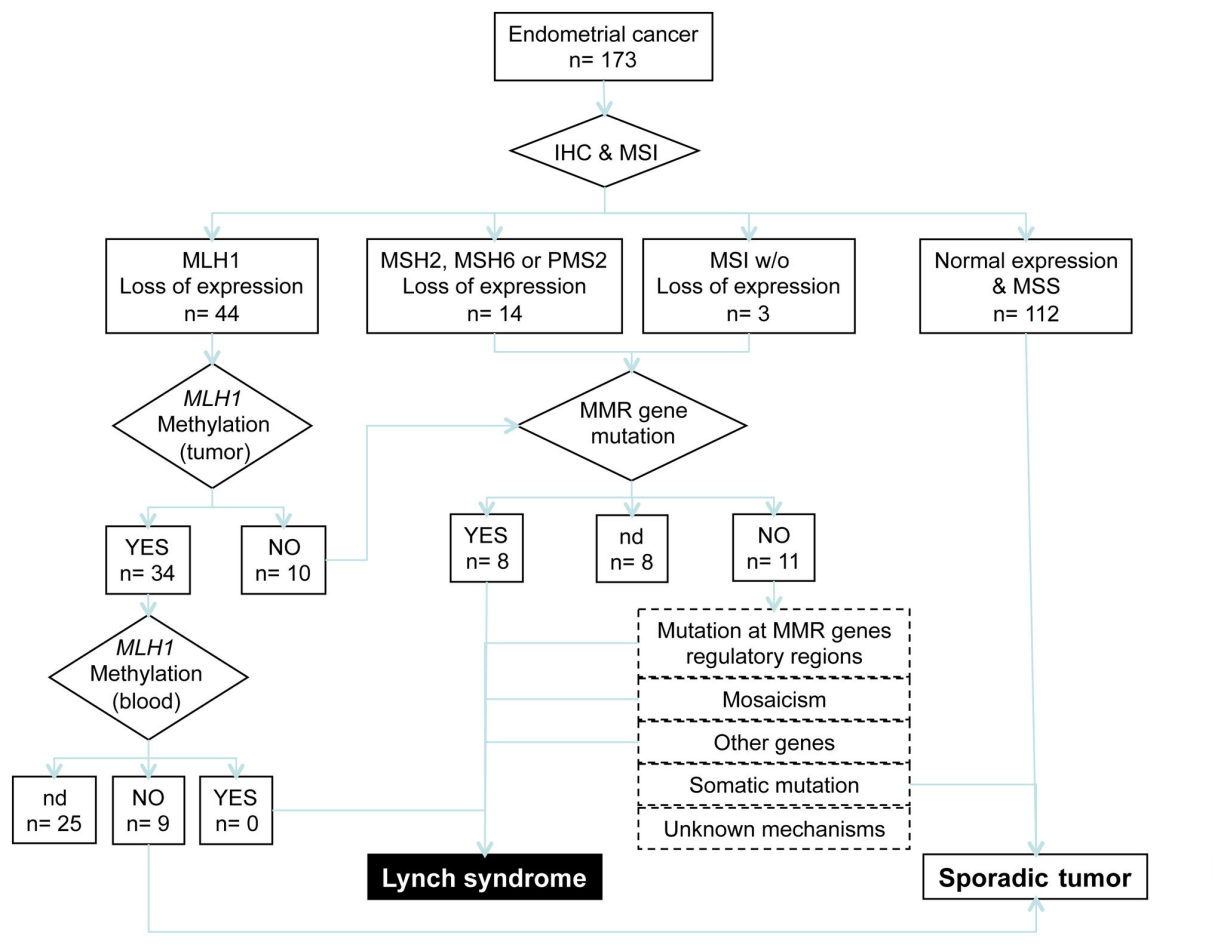
Decision–support flow diagram for patients with Lynch syndrome: genetic diagnosis strategy.

Cases with loss of MLH1 expression were tested for *MLH1* methylation in the tumor DNA. Any cases with such methylation were then analyzed for *MLH1* methylation in DNA from blood cells to identify suspected constitutional *MLH1* epimutations (Figure 1). We used the methylation-specific multiplex ligation-dependent probe-amplification technique (MS-MLPA Kit ME011; MRC-Holland, Amsterdam, The Netherlands) to study the methylation status of *MLH1*, according to the manufacturer’s protocols. The target regions for *MLH1* gene silencing by hypermethylation are located in the C and D regions of the *MLH1* promoter (from nucleotide positions -248 to -178 and -109 to +15) [21], which were tested using the probes *MLH1*-3 and -4, respectively. The mean results for these two probes were calculated to obtain the methylation ratio. The threshold for methylated versus unmethylated status was set at 15%, based on a previous study of *MLH1* gene silencing [22]. MS-MPLA fragments were analyzed using an ABI Prism 3130 Genetic Analyzer, and Genotyper software (Life Technologies).

### Germline mutations

Patients in whom we suspected LS and were candidates for genetic testing were referred to the Genetic Counseling division in our unit. Suspicion of LS was based on MMR status (IHC and MSI results) and the *MLH1* methylated status, according to our decision tree algorithm (Figure 1). *MLH1* gene testing was performed in cases where tumors showed loss of protein expression and unmethylated *MLH1*. *MSH2* mutational analysis was performed in those cases with *MSH2-*negative staining tumors. *MSH6* germline analysis was done in patients with a lack of MSH6 protein expression but with normal expression of MSH2. Tumors with a combined lack of MSH2 and MSH6 proteins with an undetected mutation in *MSH2* were also tested for *MSH6* genetic alterations. Cases tested for *MSH2* and *MSH6* with no mutation detected were also analyzed for large rearrangements at the *EPCAM* locus. Genetic testing for *PMS2* was performed only in those patients with tumors showing loss of expression of PMS2 and normal expression of MLH1.

Germline mutation studies were performed on genomic DNA isolated from peripheral blood leucocytes or from nontumorous endometrial tissue. Detection of point mutations was conducted using PCR and direct sequencing of the whole coding sequence and intron–exon boundaries for each gene [12]. Large rearrangements (deletions and/or insertions) for MMR genes were screened by MLPA according to the manufacturer protocols (Salsa MLPA kits P003, P072 and P008; MRC-Holland). Confirmation testing was also performed using MLPA with a different combination of probes (Salsa MLPA kit P248; MRC-Holland). Analysis of deletions at the *EPCAM* locus was also done using MLPA (Salsa MLPA kit P072-B1; MRC-Holland). The interpretation of genetic analysis results was based on the American College of Medical Genetics (ACMG) Recommendations for Standards for Interpretation of Sequence Variations [23], the InSiGHT database [24], and the references therein were reviewed for classifying genetic variants.

### Data management and statistical analysis

Analysis was carried out using *R* software, version 2.15.2 (The R Project for Statistical Computing. Available: http://www.r-project.org. Accessed 2013 Oct 21) and the Epicalc epidemiological analysis package [25]. Relevant measures of central tendency (means, medians and interquartile ranges for skewed data) were used to explore the data. The chi-squared test was used to compare qualitative variables. Student’s *t* test was used to compare normally distributed continuous variables. Significance was set at *p* < 0.05 and results are presented as the odds ratio (OR) and 95% confidence interval (CI).

## Results

A total of 173 unselected patients with ECs was included. The mean age at diagnosis was 63.3 years (range 29–90). All patients underwent biopsy or hysterectomy and paraffin wax-embedded tissues were available for IHC and molecular testing. The clinical and histopathology characteristics of the tumors are shown in Table 1. Most tumors were of endometrioid histology (79.2%) and FIGO grade 1 (54.9%). No myometrial invasion was found in 10.3% of patients (15/146). About 63.7% of cases had myometrial invasion ≤50% (96/146) and 26% (38/146) had myometrial invasion >50%. TILs were present in 29.4% of tumors (47/160) and 17.9% of tumors had LVI (24/134). Twenty-six patients (17.1%) had synchronous endometrial and ovarian cancers.

**Table 1:** Clinical and pathologic characteristics.

**Variable**	**N**	**[ ]**
Number of patients	173	
Mean age (SD) and range in years	63.27(+/- 12.37)	29-90
	**N**	%
**Stage at initial diagnosis**	**158**	
I	119	75.3
II	6	3.8
III	31	19.6
IV	2	1.3
**Histology**	**173**	
Endometroid	137	79.2
Poorly Diferenciated	8	4.6
Papilary Serous	13	7.5
Clear Cell	11	6.4
Mülerian Mixet Tumor	4	2.3
**Grade (FIGO)**	**173**	
1	95	54.9
2	29	16.8
3	49	28.3
**Myometrial invasion**	**146**	
None	15	10.3
≤50%	93	63.7
>50%	38	26.0
**Tumor infiltrating lymphocytes**	**160**	
No	113	70.6
Yes	47	29.4
**Lymphovascular invasion**	**134**	
No	110	82.1
Yes	24	17.9
**Low Uterin Segment**	**173**	
No	157	90.8
Yes	16	9.2
**Synchronous ovarian cancer**	**152**	
No	126	82.9
Yes	26	17.1

Familial histories of cancer, histopathology and molecular characteristics regarding LS are shown in Table 2. Eight patients (4.6%) had a history of colorectal lesions and 10 (5.8%) had a history of breast cancer and other neoplasias. A familial history of cancer was available in 87 cases: 42 (48.3%) fulfilled the rBG criteria and four (4.6%) fulfilled the AmII criteria. Family history of cancer from the remaining 86 cases was unavailable because unconfirmed family history, were lost to follow-up or deceased. 

**Table 2:** Features related to Lynch syndrome.

**Variable**	**N**	**%**
**Lynch syndrome criteria**	**87**	
Reviewed Bethesda criteria	38	43.68
Amsterdam II criteria	4	4.60
No Fullfill	45	51.72
**Personal History of Colorectal Lesions**	**8**	
Colon cancer	6	3.47*
Polyps	2	1.16*
**Personal History Others Tumors**	**15**	
Breast cancer	10	5.78*
Lung cancer	2	1.16*
Pleura	1	0.58*
Thyrod cancer (Medular)	1	0.58*
Urothelial Cancer	1	0.58*
**IHC protein expression**: **Loss of MLH1/MSH2/MSH6/PMS2**	**173**	
No	115	66.47
Yes	58	33.53
**MSI Status**	**173**	
MSS	126	72.83
MSI	47	27.17
**Mismatch Repair status**	**173**	
Proficient	112	64.74
Deficient	61	35.26

* Calculated from whole series (n=173).

We found an altered MMR picture (loss of MMR protein expression and/or MSI) in 61 patients (35.3%). Loss of MLH1 expression was found in 44 patients (25.4%). From these, 34 (77.3%) showed *MLH1* hypermethylation in the tumor. Thereafter, *MLH1* hypermethylation analysis was done in nine of these cases where DNA from blood cells was available, but gave negative results in all of them. Thus, all tumors with *MLH1* hypermethylation were considered sporadic ECs.

Loss of MSH2/MSH6 was detected in five patients (2.9%) while loss of only MSH6, or only PMS2 protein expression, was observed in eight (4.6%). One case, with both losses of MSH6 and PMS2, was found (Table S1 in File S1). All these cases, together with another 10 cases with loss of MLH1 expression and absence of *MLH1* methylation were considered suspicious of LS and suitable for genetic testing.

A significant association was found between the IHC and MSI results (*p* < 0.0001) showing concordance in 90.2% of cases (156/173). Discordances between IHC and MSI were found in 17 cases (9.8%). Loss of expression of MMR proteins and MSS was found in 14 cases. Normal expression of MMR proteins and MSI were found in three cases.

We did not have evidence for a sporadic origin of these tumors so they were also considered as suspected LS. Finally, a total of 27 (15.6%) cases were included in the genetic analysis for germline mutation screening.

Comparative analysis among the suspected cases of LS and cases with MMR proficient ECs showed that the suspected hereditary condition was more frequently found in women younger than 50 years (OR 2.84; 95% CI 1.04–7.77). No significant differences were found in any other clinical or pathological variables (Table 3). Similar results were obtained when we compared patients showing abnormal MMR with those with retained MMR function (Table S2 in File S1), with the exception of TIL and LIV, which was strongly associated with abnormal MMR tumors (OR 5.88; 95% CI 2.80–12.31 and OR 3.03; 95% CI 1.22–7.56, respectively).

**Table 3:** Comparative analysis of patients with suspected Lynch syndrome and MMR proficient endometrial cancers.

	**Suspected LS**		**MMR proficient**			p
Number of patients	27		146			
Mean age (SD)	57.70	(12.45)	64.31	(12.12)		**0.01**
	N	%	N	%	OR (IC 95%)	p
**Age**						
<50 yrs	7	25.93	16	10.96	2.84 (1.04-7.77)	**0.04**
≥50 yrs	20	74.07	130	89.04		
**Histology**						
Endometroid (type I)	22	81.48	123	84.25	0.82 (0.28-2.39)	0.72
Special (type II)	5	18.52	23	15.75		
**Grade (FIGO)**						
High	9	33.33	40	27.40	1.32 (0.5-3.19)	0.53
Low	18	66.67	106	72.60		
**Myometrial invasion**						
**(n=146)**						
>50%	6	25.00	32	26.23	0.93 (0.34-2.57)	0.90
≤50%	18	75.00	90	73.77		
**Tumor infiltrating lymphocytes**						
**(n=160)**						
Yes	9	33.33	38	28.57	1.25 (0.52-3.03)	0.62
No	18	66.67	95	71.43		
**Lymphovascular invasion**						
**(n=134)**						
Yes	7	30.43	17	15.32	2.42 (0.87-6.76)	0.09
No	16	69.57	94	84.68		
**Low Uterin Segment**						
Yes	4	14.81	12	8.22	1.94 (0.57-6.54)	0.28
No	23	85.19	134	91.78		
**Synchronous ovarian cancer**						
**(n=152)**						
Yes	5	18.52	22	17.60	1.06 (0.36-3.11)	0.91
No	22	81.48	103	82.40		
**Reviewed Bethesda criteria**						
**(n=86)**						
Fullfill	14	60.87	28	44.44	1.94 (0.73-5.14)	0.18
No Fullfill	9	39.13	35	55.56		
**Amsterdam II criteria**						
**(n=17)**						
Fullfill	3	33.33	1	12.50	3.50 (0.28-43.16)	0.31
No Fullfill	6	66.67	7	87.50		

Germline mutation analyses were performed for 19 patients of the suspected LS group (19/27, 70.4%). Genetic test results for the remaining eight patients were unavailable because they rejected the testing (3/27, 11.1%), were lost to follow-up (3/27, 11.1%) or deceased (2/27, 7.4%).

We found eight patients with pathogenic mutations (8/19, 42.1%) representing 4.6% of the whole series; one in *MLH1*, three in *MSH2*, three in *MSH6* and one in *PMS2* genes (Table 4). The mean age of these patients was 49 years, significantly lower than in the non-LS EC group (Table S3 in File S1). Twenty-five percent of them did not fulfill the rBth criteria and another 25% showed negative MSI (with isolated loss of protein expression in MSH6 and PMS2) (Table S4 in File S1). Two patients had synchronous ovarian cancers and one had a synchronous colon cancer (Table 5). The presence of synchronous ovarian cancer and tumor infiltrating lymphocytes was associated to confirmed LS cases when compared to non-LS EC (Table S3 in File S1). 

**Table 4:** IHC patterns of suspected Lynch syndrome.

**IHC pattern**	**Suspected Lynch**	**Germline mutation / analyzed cases**
Loss of MLH1 no Methylated	10	1/6
Loss of MSH2/MSH6	5	3/4
Loss of MSH6	7	2/5
Loss of PMS2	1	1/1
Loss of MSH6/PMS2	1	0/1
MSI + normal IHC	3	1/2
TOTAL	27	**8/19**

**Table 5:** Characteristics of patients with germline mutation.

Case	rBG	Age	MSI status	IHC loss	Gene	Nucleotide nomenclature	Protein nomenclature	Synchronous tumor/lesions
End131	Yes	41	MSI	MLH1/PMS2	*MLH1*	c.2154_2157insAACA^#^	p.His718Glnfs*5	Colonic Polyp
End111	Yes	45	MSI	MSH2/MSH6	*MSH2*	c.1-?_645+?del Deletion of exon 1-3	p?	Colon cancer
End091	Yes	40	MSI	MSH2/MSH6	*MSH2*	c.1226_1227delAG	p.Gln409Argfs*7	Ovarian cancer
End003	Yes	60	MSI	MSH2/MSH6	*MSH2*	c.1387-?_1661del Deletion of exon 9-10	p?	None
End014	No	61	MSI	MSH6	*MSH6*	c.2731CT	p.Arg911*	None
End088	Yes	45	MSS	MSH6	*MSH6*	c.1367GA^#^	p.Trp456*	Ovarian cancer
End137	No	56	MSI	No loss	*MSH6*	c.1367GA^#^	p.Trp456*	None
End034	Yes	44	MSS	PMS2	*PMS2*	c.538-?_705+?del Deletion of exon 6^#^	p?	None

# Not described at InSiGHT database.

Three of the mutations (37.5%) were large deletions (two in *MSH2* and one in *PMS2*), another three (37.5%) were nonsense mutations (all in the *MSH6* gene) and two (25%) were frameshift mutations (one insertion in *MLH1* and one deletion in *MSH2*). Another two patients showed genetic missense variants of unknown clinical significance, both in *MSH6* (c.116GA; p.Gly39Glu; and c.1109TC; p.Leu370Ser). Prediction using Polyphen-2 (http://genetics.bwh.harvard.edu/pph2/) classified both variants as probably pathogenic. In addition, both tumors showed loss of expression of MSH6 with conserved expression of MSH2, and absence of MSI.

In the association study comparing mutated with nonmutated cases, only age was found to be significant. Mutations were more frequently found in patients with EC diagnosed before 50 years of age (OR 16.67; 95% CI 1.01–588.03; *p* = 0.048). No significant associations were found regarding rBG criteria, IHC, MSI status, histopathology or the presence of synchronous tumors.

## Discussion

A small proportion of ECs might be the result of a genetic risk condition [3]. LS is the main syndrome involved in such cases [5,6], although the existence of a familial site-specific EC genetic entity separate from LS has been sugested [26]. The current data about the prevalence of LS among patients with EC is limited and restricted to populations from the United States [13–15]. Differences in the prevalence of genetic diseases are frequently observed between different populations, especially for syndromes where the penetrance is incomplete and other genetic and environmental factors might act as penetrance modifiers.

We aimed to determine the prevalence of LS among patients with ECs in our Spanish population, to establish proper screening strategies for identifying individuals with genetic predispositions to other tumors; consequently, to identify family members at risk and establish personalized follow-up recommendations.

We used a prevalence study using established recommendations and consensus algorithms for screening and mutation analysis [7]. To maximize our mutation detection sensitivity, no limit on age at diagnosis was considered. IHC and MSI analysis were performed for all tumors. *MLH1* methylation was tested in tumors and also in blood when required and mutational analysis was done for the four common MMR and *EPCAM* genes.

Our IHC and MSI screening detected 35.3% (61/173) of tumors with deficient MMR, which is higher than those 22.8% (124/543) and 25.3% (62/245) found by Hampel et al*,*2006 and Moline et al, 2013, respectively. We found normal IHC expression of MMR proteins together with MSI in 3/61 cases (4.9%). Similar results were obtained by Hampel et al. 2006 (6.3%, 6/96) [13]. Moreover, we found that about 23% (14/61) of tumors had loss of expression of an MMR protein and MSI. The expected percentage of loss of expression and MSI in the Hampel (2006) study [13] would be about 15%. Interestingly, we found that two unrelated patients with these apparent discordances had the same mutation at *MSH6* (c.1367GA; p.Trp456*), suggesting that in some circumstances the nature of the second hit could be determinant for the tumor’s IHC and MSI characteristics (Table 5). Our data shows that in patients with EC, up to 27.9% of cases with MMR deficiency might have either normal IHC results or MSI and reinforces the need for implementing screening with both techniques.

Comparative analysis of our MMR deficient versus positive cases did show a significant association with LVI and TILs, as described elsewhere [27,28]. However, no association was found between age at diagnosis and the rBG or AmII criteria (Table S2 in File S1). These results could be explained by the frequent occurrence of somatic MMR deficiency in ECs [13].

*MLH1* methylation and *BRAF* mutation analyses allow one to identify sporadic forms of CRC among MMR-deficient tumors. We found no case mutated in *BRAF* (data not shown). Thus, this gene does not have a major role in EC and should not be included in the screening algorithm [15]. In contrast, *MLH1* promoter methylation was present in the majority of tumors along with the loss of expression of MLH1 (77.3%, 34/44). This proportion was significantly lower than the 94% (79/84) found by Hampel et al, 2006 [13]. Differences among the two cohorts and different methodologies for the methylation analysis (MS-MLPA vs MS-PCR) might have contributed to this disparity.

Constitutional epimutations to the *MLH1* gene in cases of EC are very rare [29]. None of the nine cases analyzed with tumor methylation had *MLH1* methylation in blood DNA. Thus, a tumor with *MLH1* methylation is unlikely to be LS-associated. In contrast, a tumor that shows loss of MLH1 and PMS2 by IHC, with no evidence of methylation, is likely to be associated with LS [1]. 

After the initial screening, we selected 27 patients (15.61%) suspected of LS. The mean age in this group was 57.7 years and patients younger than 50 were more frequent (Table 3). No other pathological or clinical characteristic was found to be associated with suspected LS cases. Currently, there have been attempts to identify pathological factors in LS-associated EC. Some authors have identified the tumor localization reporting a significantly higher prevalence of lower uterine segment tumors in patients with LS [30,31]. Westin et al. concluded that screening for LS should be considered in cases with ECs originating in the lower uterine segment [30]. This location can be a source of diagnostic consternation because it can host both endometrial and endocervical carcinomas, resulting in tumor misclassification [32].

Mutations were found in eight patients (42.1%) with a mean age of 49 years. *MSH6* and *MSH2* were mutated in six cases (Table 5). Two cases with mutations *MSH6* (ages 56 and 61 years) did not fulfill the rBG criteria and no other synchronous tumor was present at diagnosis. Thus, about 25% of patients with EC associated with LS might appear to have sporadic tumors and go undiagnosed when the rBG criteria are used to test a suspicion of LS. LS-related ECs commonly result from mutations in *MSH6* and occur at later ages than do mutations in *MLH1* and *MSH2* [33,34]. For patients with LS-related ECs, the risk of developing a second cancer following the initial EC diagnosis is estimated at 25% in 10 years and 50% at 15 years [35,36]. The 20-year cumulative risk of cancer after endometrial cancer has been recently reported with 48% risk for CRC; 11% for cancer of the kidney, renal pelvis, or ureter; 9% for urinary bladder cancer; and 11% for breast cancer [37]. Among patients with LS, 50% of the ECs present before a diagnosis of CRC, if the diagnoses are not synchronous. Therefore, EC can serve as a ‘sentinel’ cancer for patients and potentially for their family members [38].

In our study, mutations were found more frequently in patients with ECs diagnosed before 50 years of age and no association was found regarding the rBG criteria, IHC or MSI status, histopathology and the presence of synchronous tumors. However, it is possible that the limited sample size could be hiding any other association.

There are several possibilities to explain those cases with undetected germline mutations. First, presence of mutations at untested regulatory regions of the analyzed genes; second, an MMR deficiency caused by somatic biallelic inactivation; third, genetic mosaicism; fourth, germline mutations in other genes directly or indirectly involved with MMR function, such as *SETD2* [39], *POLE* and *POLD1* [40], and finally other unknown genetic or epigenetic mechanisms (Figure 1).

Many institutions and policy groups are considering whether to implement screening for patients with LS among patients with ECs. Different screening strategies for women with EC have been shown to be cost effective [41,42]. IHC triage of all cases of EC can identify most mutation carriers but at considerable cost. The inclusion of at least one first-degree relative with an LS-associated cancer at any age might prove cost effective [42]. Nevertheless, it is important to stress that multiple local factors might interfere in the efficiency of any process. New approaches for making the universal screening of patients with EC by IHC more cost effective are in progress. Recent data suggest that a two-antibody panel testing for PMS2 and MSH6 is as effective as the four-antibody panel for detecting MMR abnormalities [43,44]. We observed that to increase sensibility in LS diagnosis an analysis of MSI combined with IHC should be considered because about 5% (3/61) of patients with suspected LS and one-eighth of confirmed LS cases in our study had MSI with intact IHC.

The prevalence of LS we found among patients with EC was 4.6% (8/173); with a predictive frequency of 6.6% for the Spanish population. This prediction was made extrapolating the found frequency of mutated cases and considering the possible absence of lost cases. Is important to note that the present work is a prevalence study conducted in a single hospital from Spain, therefore extrapolation of data to the whole Spanish population may be biased.

Previous studies from North American populations showed prevalence rates ranging from 1.8% to 4.5% [13–15]. In our population, we found that the prevalence of LS among patients with EC is sixfold higher than the prevalence of LS among patients with CRCs (4.6% vs 0.7%) [12].

In conclusion, we found a high prevalence of LS among patients with ECs (4.6–6.6%). Unlike CRC, only the patient’s age at diagnosis was found to be associated with LS. Consistent with these results, we consider that universal screening of all patients with ECs by IHC, MSI and *MLH1* methylation analysis should be recommended.

## Supporting Information

File S1 **Supporting information**.
Table S1, Result of the pre-genetic test screening. Table S2, Factors associated with mismatch repair profile. Table S3, Comparative analysis of patients with Lynch syndrome and non-LS endometrial cancers. Table S4, Lynch syndrome related variables and Bethesda guidelines.
(DOC)
