# Supplementary material for: Prevalence of Lynch Syndrome among Patients with Newly Diagnosed Endometrial Cancers
Source: PLoS One. 2013 Nov 7;8(11):e79737. doi: 10.1371/journal.pone.0079737 (PMC3820559; doi:10.1371/journal.pone.0079737)
Supplement: File S1 — Supporting information. Table S1, Result of the pre-genetic test screening. Table S2, Factors associated with mismatch repair profile. Table S3, Comparative analysis of patients with Lynch syndrome and non-LS endometrial cancers. Table S4, Lynch syndrome related variables and Bethesda guidelines. (DOC) [file pone.0079737.s001.doc]

Table S1. Result of the pre-genetic test screening.

| **Immunohistochemistry expression** | N | % |
| --- | --- | --- |
| Normal expression | 115 | 66.47 |
| Loss only of MSH6 | 7 | 4.05 |
| Loss only of PMS2 | 1 | 0.58 |
| Loss of MLH1/PMS2 | 42 | 24.28 |
| Loss of MSH2/MSH6 | 5 | 2.89 |
| Loss of MSH6/PMS2 | 1 | 0.58 |
| Loss of MLH1/PMS2/MSH6 | 2 | 1.16 |
|  |  |  |
| **MMR IHC and MSI analysis** |  |  |
| Normal expression and MSS | 112 | 64.74 |
| MMR loss of expression and MSI | 44 | 25.43 |
| Normal expression and MSI | 3 | 1.73 |
| MMR loss of expression and MSS | 14 | 8.09 |
|  |  |  |
| **Methylation Analysis** | N | % |
| MLH1 loss expression and Metylated | 34 | 77.27 |
| MLH1 loss expression, non Metylated | 10 | 22.73 |

Table S2. Factors associated with mismatch repair profile

|  | **MMR(+)** | | **MMR(-)** | |  | p |
| --- | --- | --- | --- | --- | --- | --- |
| Number of patients | 61 |  | 112 |  |  |  |
| Mean age (SD) | 61.85 | (12.26) | 64.05 | (12.42) |  | 0.26 |
|  | N | % | N | % | OR (IC 95%) | p |
| **Age** |  |  |  |  |  |  |
| <50 yrs | 10 | 16.39 | 13 | 11.61 | 1.49 (0.61-3.63) | 0.38 |
| ≥50 yrs | 51 | 83.61 | 99 | 88.39 |  |  |
| **Histology** |  |  |  |  |  |  |
| Endometroid (type I) | 53 | 86.89 | 92 | 82.14 | 1.44 (0.59-3.49) | 0.42 |
| Special (type II) | 8 | 13.11 | 20 | 17.86 |  |  |
| **Grade (FIGO)** |  |  |  |  |  |  |
| High | 21 | 34.43 | 28 | 25 | 1.57 (0.79-3.10) | 0.19 |
| Low | 40 | 65.57 | 84 | 75 |  |  |
| **Myometrial invasion (n=146)** | |  |  |  |  |  |
| >50% | 13 | 22.81 | 25 | 28.09 | 0.76 (0.34-1.63) | 0.48 |
| ≤50% | 44 | 77.19 | 64 | 71.91 |  |  |
| **Tumor infiltrating lymphocytes (n=160)** | | |  |  |  |  |
| Yes | 31 | 52.54 | 16 | 15.84 | 5.88 (2.80-12.31) | **<0.001** |
| No | 28 | 47.46 | 85 | 84.16 |  |  |
| **Lymphovascular invasión (n=134)** | | |  |  |  |  |
| Yes | 15 | 27.78 | 9 | 11.25 | 3.03 (1.22-7.56) | **0.01** |
| No | 39 | 72.22 | 71 | 88.75 |  |  |
| **Low Uterin Segment** |  |  |  |  |  |  |
| Yes | 7 | 11.48 | 9 | 8.04 | 1.48 (0.52-4.2) | 0.46 |
| No | 54 | 88.52 | 103 | 91.96 |  |  |
| **Synchronous ovarian cáncer (n=152)** | | |  |  |  |  |
| Yes | 11 | 18.33 | 16 | 17.39 | 1.06 (0.46-2.48) | 0.88 |
| No | 49 | 81.67 | 76 | 82.61 |  |  |
| **Bethesda Reviewed criteria (n=86)** | | |  |  |  |  |
| Fullfill | 22 | 56.41 | 20 | 42.55 | 1.74 (0.74-4.11) | 0.20 |
| No Fullfill | 17 | 43.59 | 27 | 57.45 |  |  |
| **Amsterdam II criteria (n=17)** | |  |  |  |  |  |
| Fullfill | 4 | 30.77 | 0 | 0 | 1.44 (1.0-2.07) | 0.20 |
| No Fullfill | 9 | 69.23 | 4 | 100 |  |  |

Table S3. Comparative analysis of patients with Lynch syndrome and non-LS endometrial cancers

|  | LS | | Non-LS | |  | p |
| --- | --- | --- | --- | --- | --- | --- |
| Number of patients | 8 |  | 165 |  |  |  |
| Mean age (SD) | 48.75 | (8.31) | 63.98 | (12.12) |  | **0.001** |
|  | N | % | N | % | OR (IC 95%) | p |
| **Age** |  |  |  |  |  |  |
| < 50 years old | 5 | 62.50 | 18 | 10.91 | 13.61(2.99,61.78) | **0.0001** |
| ≥50 years old | 3 | 37.50 | 147 | 89.09 |  |  |
| **Histology** |  |  |  |  |  |  |
| Endometroid (type I) | 7 | 87.50 | 138 | 83.64 | 1.37(0.16,11.58) | 0.77 |
| Special (type II) | 1 | 12.50 | 27 | 16.36 |  |  |
| **Grade (FIGO)** |  |  |  |  |  |  |
| High | 3 | 37.50 | 46 | 27.88 | 1.55 (0.36, 6.76) | 0.55 |
| Low | 5 | 62.50 | 119 | 72.12 |  |  |
| **Myometrial invasion (n=146)** | |  |  |  |  |  |
| >50% | 1 | 14.29 | 37 | 26.62 | 0.46(0.05, 3.96) | 0.47 |
| ≤50% | 6 | 85.71 | 102 | 73.38 |  |  |
| **Tumor infiltrating lymphocytes (n=160)** | | |  |  |  |  |
| Yes | 5 | 62.50 | 42 | 27.63 | 4.37 (0.99, 19.08) | **0.035** |
| No | 3 | 37.50 | 110 | 72.37 |  |  |
| **Lymphovascular invasion (n=134)** | |  |  |  |  |  |
| Yes | 3 | 37.50 | 21 | 16.67 | 3.0 (0.67, 13.53) | 0.14 |
| No | 5 | 62.50 | 105 | 83.33 |  |  |
| **Low Uterin Segment** | |  |  |  |  |  |
| Yes | 2 | 25.00 | 14 | 8.48 | 3.60 (0.66, 19.51) | 0.12 |
| No | 6 | 75.00 | 151 | 91.52 |  |  |
| **Synchronous ovarian cancer (n=152)** | | |  |  |  |  |
| Yes | 4 | 50.00 | 23 | 15.97 | 5.26(1.23, 22.56) | **0.014** |
| No | 4 | 50.00 | 121 | 84.03 |  |  |
| **Family history (rBG + AmII) (n=87)** | |  |  |  |  |  |
| Fullfill | 6 | 75.00 | 36 | 45.57 | 3.58 (0.68, 18.85) | 0.11 |
| No Fullfill | 2 | 25.00 | 43 | 54.43 |  |  |

Table S4. Lynch syndrome related variables and Bethesda guidelines.

|  | rBG Fullfill | | rBG not Fullfilling | |  |  |
| --- | --- | --- | --- | --- | --- | --- |
| Variable | N | % | N | % | OR (95% CI) | p |
| **IHC** |  |  |  |  |  |  |
| Loss | 22 | 52.38 | 14 | 31.82 | 2.35 (0.98-5.67) | 0.053 |
| Normal | 20 | 47.62 | 30 | 68.18 |  |  |
| **MSI status** |  |  |  |  |  |  |
| Unstable | 16 | 38.10 | 16 | 36.36 | 1.08 (0.45-2.58) | 0.868 |
| Stable | 26 | 61.90 | 28 | 63.64 |  |  |
| **Lynch Suspected** |  |  |  |  |  |  |
| Yes | 14 | 33.33 | 9 | 20.45 | 1.94 (0.73-5.14) | 0.177 |
| No | 28 | 66.67 | 35 | 79.55 |  |  |
| **Mutation** |  |  |  |  |  |  |
| Yes | 6 | 50 | 2 | 28.57 | 2.00 (0.26-5.38) | 0.502 |
| No | 6 | 50 | 5 | 71.43 |  |  |
